# Deep learning segmentation of periarterial and perivenous capillary-free zones in optical coherence tomography angiography

**DOI:** 10.1117/1.JBO.30.5.056005

**Published:** 2025-05-08

**Authors:** Mansour Abtahi, Behrouz Ebrahimi, Albert K. Dadzie, Mojtaba Rahimi, Srishti Kolla, Yi-Ting Hsieh, Michael J. Heiferman, Jennifer I. Lim, Xincheng Yao

**Affiliations:** a University of Illinois Chicago, Department of Biomedical Engineering, Chicago, Illinois, United States; b University of Illinois Chicago, Department of Ophthalmology and Visual Sciences, Chicago, Illinois, United States; c National Taiwan University Hospital, Department of Ophthalmology, Taipei, Taiwan

**Keywords:** capillary-free zones, optical coherence tomography angiography, diabetic retinopathy, deep learning

## Abstract

**Significance:**

Automated segmentation of periarterial and perivenous capillary-free zones (CFZs) in optical coherence tomography angiography (OCTA) can significantly improve early detection and monitoring of diabetic retinopathy (DR), a leading cause of vision impairment, by identifying subtle microvascular changes.

**Aim:**

We aimed to develop and evaluate deep learning models, including convolutional neural networks (CNNs) and vision transformers (ViTs), for precise segmentation of periarterial and perivenous CFZs. Quantitative features derived from the segmented CFZs were assessed as potential biomarkers for DR.

**Approach:**

OCTA images from healthy controls, patients with diabetes but no DR (NoDR), and those with mild DR were utilized. Automated CFZ maps were generated using deep learning models such as UNet, UNet++, TransUNet, and Segformer. Quantitative features, including CFZ ratios, counts, and mean sizes, were analyzed to characterize disease progression.

**Results:**

UNet++ with EfficientNet-b7 achieved the best performance, with a mean intersection over union of 86.48% and a Dice coefficient of 89.87%. Quantitative analyses revealed significant differences in CFZ metrics between the control, NoDR, and mild DR groups, demonstrating their potential as sensitive biomarkers for early DR detection and monitoring.

**Conclusions:**

The study underscores the efficacy of deep learning models in automating CFZ segmentation and introduces quantitative features as biomarkers for DR. These findings support further exploration of CFZ analysis in retinal disease diagnostics and therapeutic monitoring.

## 1 Introduction

Early detection of retinal diseases and effective monitoring of treatment responses rely on quantitative analysis of key biomarkers, including microvascular changes in the retina. Various diseases, such as diabetic retinopathy (DR), strokes (cerebrovascular accidents), hypertensive retinopathy, and different vascular disorders, can cause alterations in retinal vasculature.^1^^,^^2^ DR, in particular, primarily affects microvasculature, leading to capillary level changes detectable with advanced imaging techniques. Optical coherence tomography angiography (OCTA) provides a noninvasive way to visualize retinal capillary networks with micrometer-level resolution, enabling the precise assessment of retinal neurovascular changes.^3^ A growing body of research^4^^–^^12^ has focused on the quantitative analysis of OCTA images to assess microvascular alterations and objectively classify retinal diseases. These studies have highlighted the effectiveness of using OCTA-based metrics to detect subtle changes in the retinal microvasculature, thus providing a refined approach to early diagnosis and disease stratification. Efforts to distinguish arteries and veins in OCTA imaging have refined retinal disease classification, as these vessels show distinct pathological changes.^13^^,^^14^ Advanced machine learning techniques have improved their automated segmentation, enhancing diagnostic precision and providing insights into specific vascular alterations in DR.^15^^–^^21^

In addition to arterial and venous analysis, recent works have underscored the importance of analyzing periarterial and perivenous capillary-free zones (CFZs) in detecting early microvascular changes. Periarterial or perivenous CFZs, areas of physiological avascularization,^22^ serve as a crucial clinical marker and quantitative biomarker for assessing tissue oxygenation. Larger periarterial or perivenous CFZs indicate a higher risk of ischemia and nonperfusion in DR and other retinal conditions, highlighting their potential to detect early microvascular dysfunction in the retina.^22^

Li et al. utilized graders to measure CFZ dimensions and demonstrated that periarterial CFZ dimensions in cases with severe non-proliferative diabetic retinopathy (NPDR) are significantly larger than those in healthy eyes, indicating their potential as a clinical marker for retinal microcirculation and oxygenation.^23^ Arthur et al. demonstrated that CFZs undergo remodeling with normal aging, showing significant changes in both periarterial and perivenous CFZs.^22^^,^^24^ In another study, they found that mid-peripheral CFZs were significantly enlarged in older adults at high risk for Alzheimer’s disease (AD) compared with low-risk individuals, highlighting the potential of periarterial CFZs as biomarkers for early AD detection.^25^ Tang et al. showed that periarterial CFZs were significantly enlarged in eyes with branch retinal vein occlusion (BRVO) compared with healthy eyes, with these changes correlating to retinal ischemia and visual function.^26^ Another study by the same group revealed that anti-VEGF therapy led to a significant reduction in periarterial CFZ areas in BRVO patients, with smaller baseline periarterial CFZ sizes predicting better visual outcomes over time.^27^ All these studies highlight the potential of periarterial and perivenous CFZ analysis as valuable biomarkers for monitoring disease progression and therapeutic response in retinal vascular conditions. Automated and precise segmentation of these CFZs would allow for large-scale, objective, and reproducible assessment of microvascular health, enabling clinicians to detect subtle, early signs of ischemia and capillary dropout in DR and other pathologies. This capability could significantly enhance early diagnosis, risk stratification, and longitudinal monitoring. However, none of the aforementioned studies developed an automated method for segmenting periarterial and perivenous CFZs, and most analyses remain limited to first-order or second-order large vessels.

In this study, we present a deep learning approach for the automated segmentation of periarterial and perivenous CFZs using deep learning techniques applied to OCTA images. We developed and evaluated multiple state-of-the-art architectures, ranging from convolutional neural networks (CNNs) to vision transformers (ViTs), to achieve high-precision segmentation of these CFZs. The models were validated using a five-fold cross-validation approach, employing intersection over union (IoU), Dice coefficient, and segmentation accuracy as the primary evaluation metrics. By generating detailed CFZ maps, we aim to provide quantitative features that can enhance the assessment of retinal microvascular changes across different stages of DR. This comprehensive analysis of CFZ ratios, counts, and mean sizes establishes these features as potential biomarkers for early detection and monitoring of DR progression, ultimately paving the way for more reliable identification and classification of early-stage retinal diseases, leading to more effective clinical decision-making and therapeutic interventions.

## 2 Methods

### 2.1 Data Acquisition

This research obtained ethical approval from the Institutional Review Board (IRB) at the University of Illinois Chicago (UIC) and adhered to the ethical principles outlined in the Declaration of Helsinki. *En face* OCTA images, 6 mm × 6 mm scans, were collected between February 2017 and August 2024 at both UIC and the National Taiwan University Hospital (NTUH). A data-sharing agreement was established between UIC and NTUH. The training dataset, which consists of 80 OCTA images and their ground truths (68 control and 12 mild DR scans), is planned to be used for training and validation of the model. The test dataset, which consists of 218 OCTA images without ground truths (83 eyes from 59 control participants, 82 eyes from 48 NoDR patients, and 53 eyes from 39 mild DR patients), is planned to be used for qualitative testing of the model and quantitative analysis with the focus on early detection of DR. Each eye was scanned once using a 6 mm × 6 mm *en face* OCTA protocol. Table 1 summarizes the demographic characteristics of participants in the training and test datasets. Control subjects and diabetic patients without DR and patients with mild DR were recruited from the UIC retina clinic and NTUH. Inclusion criteria encompassed individuals aged 18 years or older diagnosed with type II diabetes mellitus. Exclusion criteria comprised individuals with macular edema, proliferative DR (PDR), prior vitrectomy surgery, a history of ocular disorders other than DR, the presence of dense cataracts or high refractive errors, and OCT images that were ungradable or had a quality index below 6. Three board-certified retina specialists (Y.H., M.J.H., J.I.L.) classified patients as NoDR or mild NPDR stage based on the Early Treatment Diabetic Retinopathy Study (ETDRS) criteria based on clinical findings derived from fundus photography as the gold standard for diagnosing DR. All patients underwent a comprehensive anterior and dilated posterior segment examination. Control OCTA images were obtained from healthy volunteers who provided informed consent for OCT/OCTA imaging. Deidentified diabetic datasets were acquired for retrospective analysis. The IRB waived the requirement for patient informed consent while adhering to patient privacy and confidentiality guidelines outlined by the IRB.

**Table 1:** Demographics of healthy subjects, NoDR, and mild NPDR patients.

	Healthy subjects	NoDR	Mild NPDR
Training dataset			
Number of subjects (n)	40	—	12
Number of images (n)	68	—	12
Age (years)	52.4 ± 16.22	—	60.83 ± 13.41
Age range	26 to 80	—	24 to 79
Sex (male/female)	39/29	—	5/7
Test dataset			
Number of subjects (n)	59	48	39
Number of images (n)	83	82	53
Age (years)	57.4 ± 14.20	61.40 ± 13.39	61.30 ± 12.44
Age range	37 to 88	33 to 86	25 to 78
Sex (male/female)	40/43	34/48	27/26

Spectral-domain (SD) *en face* OCTA data were obtained using AngioVue SD-OCT devices (Optovue, Fremont, CA, United States) at both the UIC retina clinic and NTUH. The OCT devices operated at a rate of 70,000 A-scans per second, providing an axial resolution of ∼5  μm and a lateral resolution of about 15  μm for 6  mm×6  mm scans. For this study, only superficial OCTA images segmented at the level of the retinal nerve fiber layer and the ganglion cell layer were utilized. After image reconstruction, de-identified *en face* OCTA images were extracted from the ReVue software interface (Optovue) for subsequent processing.

### 2.2 Generating Ground Truths

The CFZ refers to regions of physiological avascularization that naturally occur adjacent to arteries and veins in the retina. These areas lack capillaries under normal conditions and are identifiable as distinct spaces surrounding large vessels in OCTA images. In DR and other retinal vascular diseases, CFZs can become pathologically enlarged due to microvascular alterations and nonperfusion, reflecting early signs of ischemia. These changes, particularly in the periarterial and perivenous CFZs, manifest as irregularities that expand as retinal damage progresses. To accurately quantify these changes in *en face* OCTA images, it is essential to segment the CFZ automatically using deep learning models. The training of such models necessitates generating ground truth data for CFZ segmentation within the dataset.

**Fig. 1:**
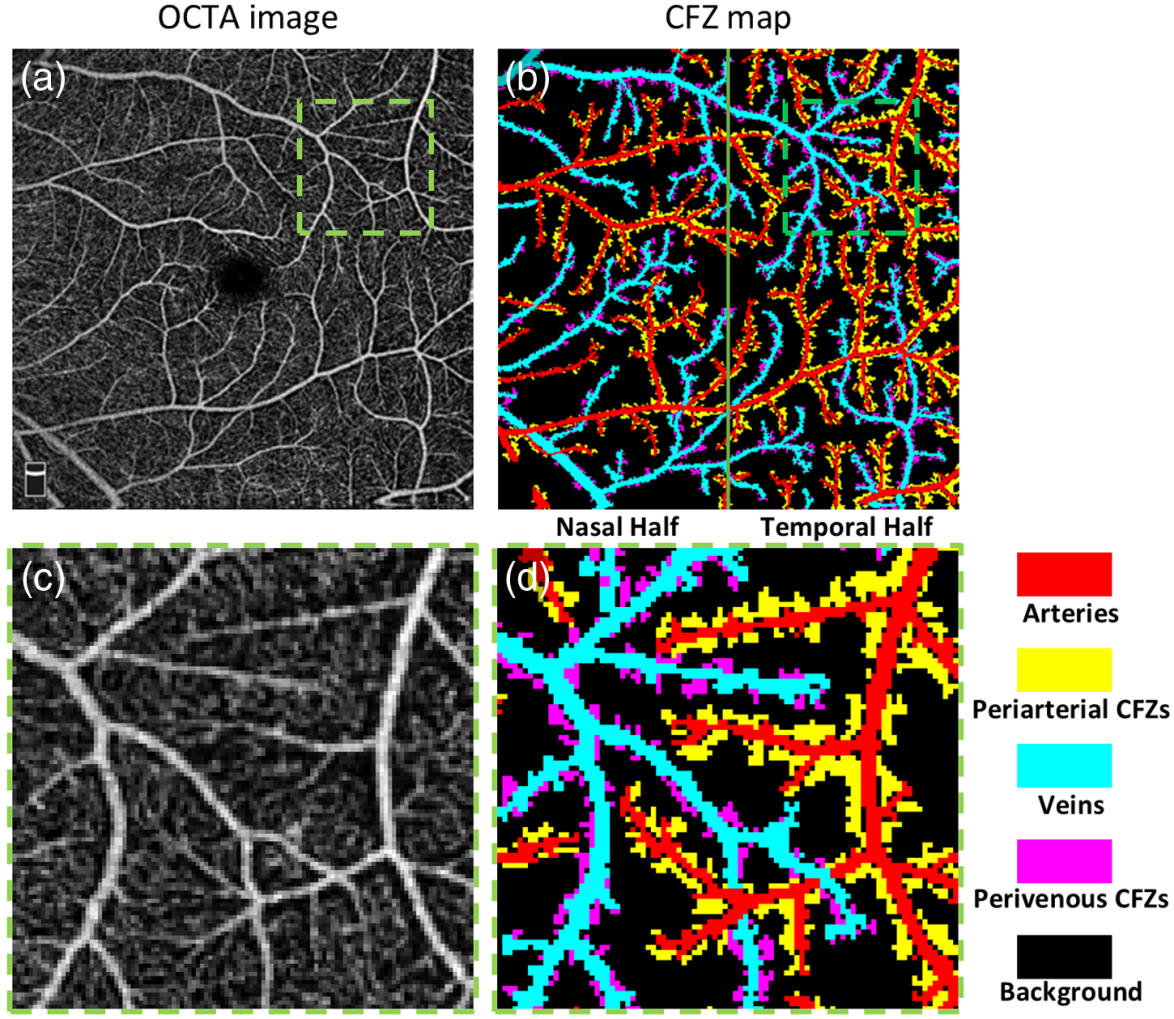
(a) OCTA image. (b) CFZ map. (c) Enlarged illustration of the green window region in (a). (d) Enlarged illustration of the green window region in panel (b).

As reported in previous publications,^19^^,^^20^ readers can rely on various characteristics in OCTA images to manually detect arteries and veins accurately in the 6  mm×6  mm dataset. Following the identification of large vessels as arteries and veins, the segmentation of the periarterial and perivenous CFZ was performed using a semi-automated image processing pipeline. The process involved the following steps: (1) the original OCTA images were preprocessed with Gaussian filtering to slightly reduce noise, (2) adaptive thresholding was applied to generate a binary mask, distinguishing avascular areas, (3) a mask was generated to identify potential CFZs using connected component analysis, (4) only areas adjacent to arteries or veins were included, and (5) manual modification of the segmented areas ensured that only the clinically relevant periarterial and perivenous CFZs were retained. We chose one expert grader for this process to ensure consistency across all images. They were reviewed and approved by experienced ophthalmologists. Figures 1(a) and 1(b) show a representative OCTA image and corresponding generated CFZ map, with enlarged areas displayed in Figs. 1(c) and 1(d).

### 2.3 Quantitative Features

As demonstrated in Fig. 1(b), we present the CFZ map containing segmentations for all arteries, veins, periarterial CFZ, and perivenous CFZ, which, to the best of our knowledge, are introduced for the first time by this paper. The CFZ map displays all vessels of varying orders and scales that are visible in the OCTA images, identifying them as arteries or veins, along with the corresponding periarterial CFZ and perivenous CFZ regions. The OCTA layer indicator area at the bottom left corner of the OCTA image Fig. 1(a) is excluded from the CFZ map as presented in Fig. 1(b).

Using the CFZ maps, we can perform a quantitative analysis across control, NoDR, and mild DR stages. The areas of periarterial or perivenous CFZ, as well as the total vasculature, can be quantified using these maps. As three quantitative features, the ratios of the periarterial or perivenous CFZ areas to the corresponding arterial or venous vessel areas, as well as the ratio of the total CFZ area to the total vasculature area, can be defined as the periarterial CFZ ratio (Ra), perivenous CFZ ratio (Rv), and total CFZ ratio (Rt), respectively, as follows:

$$R_{a}=\frac{\text{Periarterial}\;\mathrm{CFZ}\;\text{Area}}{\text{Arterial Vessel Area}},$$ (1)

$$R_{v}=\frac{\text{Perivenous}\;\mathrm{CFZ}\;\text{Area}}{\text{Venous Vessel Area}},$$ (2)

$$R_{t}=\frac{\text{Total}\;\mathrm{CFZ}\;\text{Area}}{\text{Total Vasculature Area}},$$ (3)

where the periarterial or perivenous CFZ area and arterial or venous vessel area are calculated by counting the number of pixels belonging to the corresponding area in the CFZ map. The total CFZ area is defined as the summation of the periarterial and perivenous CFZ areas, whereas the total vasculature area is the summation of the arterial and venous vessel areas. In addition, a connected components analysis can be conducted to quantify the number of CFZs and their sizes. In the connected components analysis, CFZs with a size of two pixels or less are excluded to avoid counting noise or artifacts. This threshold was chosen based on the image resolution and our empirical observation that single-pixel or two-pixel regions typically correspond to negligible voids or background noise. The number of remaining CFZs for periarterial, perivenous, or total CFZs are then defined as $N_{a}$, $N_{v}$, and $N_{t}$, respectively. The mean size of the corresponding CFZs is calculated as $M_{a}$, $M_{v}$, and $M_{t}$, which are formulated as

$$M_{a}=\frac{\sum_{i=1}^{N_{a}}\text{Area of}\;{\mathrm{pa}\mathrm{CFZ}}_{i}}{N_{a}},$$ (4)

$$M_{v}=\frac{\sum_{i=1}^{N_{v}}\text{Area of}\;{\mathrm{pv}\mathrm{CFZ}}_{i}}{N_{v}},$$ (5)

$$M_{t}=\frac{\sum_{i=1}^{N_{t}}\text{Area of}\;{\mathrm{tCFZ}}_{i}}{N_{t}},$$ (6)

where paCFZ, pvCFZ, and tCFZ represent the periarterial CFZ, perivenous CFZ, and total CFZ, respectively, the area of each CFZ is determined by counting the number of pixels in each connected component. These metrics provide additional insights into the distribution and characteristics of CFZs in the retina.

We defined nine previously unreported quantitative OCTA features ($R_{a}$, $R_{v}$, $R_{t}$, $N_{a}$, $N_{v}$, $N_{t}$, $M_{a}$, $M_{v}$, and $M_{t}$) related to CFZ maps that can be used to quantitatively analyze diseases at different stages. We are going to evaluate these quantitative features in control, NoDR, and mild DR groups.

### 2.4 Statistical Analyses

For statistical analysis of quantitative features, each eye was considered a unique observation for subjects with images of both eyes. We employed $\chi^{2}$ tests to evaluate the distribution of sex among various groups. To assess the normality of age distribution in different groups as presented with mean and standard deviation in Table 1, we conducted the Shapiro–Wilk test. Comparisons of age distribution were carried out using analysis of variance (ANOVA). To perform one-on-one comparisons of quantitative features between different groups, we utilized the Mann-Whitney U test, a non-parametric method suitable for data not assumed to follow a normal distribution. Accordingly, we present our findings using median, mean, and interquartile range (IQR) in boxplots. In all comparisons, statistical significance was determined with a p value<0.05. However, given the presence of three pairs of comparisons, the Bonferroni-corrected threshold for multiple simultaneous group comparisons can be considered as a p value of <0.017.

### 2.5 Deep Learning Models Architecture

The input to the models consists of grayscale OCTA images, which encapsulate critical information regarding blood flow strength and vessel geometry. The segmentation task involves five distinct classes: arteries, veins, periarterial CFZs, perivenous CFZs, and background, making this a multiclass segmentation problem. In this study, we evaluated various state-of-the-art deep learning architectures, including both CNNs and ViTs, for the segmentation of CFZs in OCTA images. After thorough experimentation and analysis, we present the performance of the models that demonstrated the highest efficacy in our segmentation tasks.

#### 2.5.1 UNet with EfficientNet-b7 backbone

The original UNet architecture^28^ is a widely used CNN model in biomedical image segmentation, characterized by its symmetric encoder–decoder structure and skip connections that help preserve spatial information throughout the network. In this setup, the EfficientNet-b7^29^ backbone, also a CNN, serves as the encoder, extracting rich features that the UNet decodes into precise segmentation maps. This combination leverages the strengths of both EfficientNet-b7’s feature extraction and UNet’s effective spatial information handling.

#### 2.5.2 UNet++ with EfficientNet-b7 backbone

UNet++ is an advanced extension of the original UNet architecture, designed to improve segmentation accuracy by incorporating dense skip connections and a more sophisticated network topology.^30^ These enhancements facilitate better feature fusion and representation, particularly in complex segmentation tasks. Similar to its integration with UNet, EfficientNet-b7 is employed as the backbone in this model, utilizing its compound scaling method to optimally balance network depth, width, and resolution. This setup allows EfficientNet-b7 to efficiently extract multi-scale features, which are then further refined and segmented by the UNet++ CNN architecture, resulting in precise and accurate segmentation outcomes.

#### 2.5.3 TransUNet

TransUNet is an innovative architecture that integrates ViT modules into the traditional UNet framework.^31^ The inclusion of transformers allows the model to capture long-range dependencies and contextual information across the entire image, which is a significant advantage in medical image analysis. The combination of the convolutional layers of UNet and the attention mechanisms of transformers in TransUNet provides a robust architecture capable of both local and global feature extraction.

#### 2.5.4 Segformer with Mix Transformer (MiT)-B5 encoder

Segformer is a transformer-based ViT architecture designed for efficient and scalable image segmentation.^32^ It employs the MiT-B5 backbone, which integrates the strengths of transformer networks with lightweight design principles to achieve a balance between performance and computational efficiency. This architecture is particularly well-suited for scenarios that demand both high segmentation accuracy and computational efficiency. The Segformer model excels in capturing global context, whereas its lightweight nature ensures the flexibility and efficiency required for real-time applications.

### 2.6 Loss Function

In this study, the deep learning architectures were trained using IoU loss^33^ or Jaccard loss to directly optimize the IoU score, the most commonly used evaluation metric in segmentation.^34^ For multiclass segmentation, it is defined by

$$L_{\mathrm{IoU}}=1-\frac{\sum_{c=1}^{C}\sum_{i=1}^{N}g_{i}^{c}s_{i}^{c}}{\sum_{c=1}^{C}\sum_{i=1}^{N}(g_{i}^{c}+s_{i}^{c}-g_{i}^{c}s_{i}^{c})},$$ (7)

where $g_{i}^{c}$ is the ground truth binary indicator of class label c of voxel i and $s_{i}^{c}$ is the corresponding predicted segmentation probability. N is the number of voxels in the image, and C is the number of classes.

### 2.7 Training Process

The deep learning architectures in this study were trained using the Adam optimizer with a learning rate of 0.0005 ($\beta_{1}=0.9$, $\beta_{2}=0.999$, $\epsilon =10^{-8}$) to ensure a smooth learning curve on the validation dataset. The learning rate was selected based on an empirical evaluation to ensure a smooth and fast learning curve on the validation dataset. A learning rate scheduler was used to adjust the learning rate during training, reducing it by a factor of 0.8 every 150 epochs to ensure stable convergence. The models were trained using the IoU loss function. The mini-batch size was fixed at 15, determined by the maximum capacity of the available GPU memory while maintaining training efficiency. Training continued for up to 1000 epochs. One epoch was defined as an iteration over all training batches. To mitigate overfitting, data augmentation techniques were applied on the fly during training, including random horizontal flipping, random zooming, rotation, image shifting, shearing, and brightness adjustments. Given the diverse tree-such as patterns of retinal vessels in OCTA images, the variation in vessel diameters, and the differences in image quality and location from both right and left eyes, these augmentation methods were crucial for enhancing the generalization capability of the models when segmenting unseen images.

Due to the limited dataset, a five-fold cross-validation procedure was employed, with data shuffling based on the patient’s eye. In each fold, the models were trained on 80% of the images, and evaluation was conducted on the remaining 20%. For performance evaluation, in addition to pixel-wise segmentation accuracy—defined as the proportion of correctly classified pixels (true positives + true negatives divided by the total pixels)—the IoU and Dice coefficient metrics, also known as the Jaccard Index and F1 score, respectively, are also considered. These metrics were computed by comparing the CFZ segmentation maps predicted by different models, including UNet, UNet++, TransUNet, and Segformer, against the manually labeled ground truths.

The training was conducted on a Windows 10 machine equipped with NVIDIA Quadro RTX 6000 graphics processing units (GPU). The models were implemented and evaluated using Python (v3.9) with PyTorch (v2.2.1+cu118) and CUDA (v11.8) for GPU acceleration. Training each fold of the models took ∼5  h with the aforementioned resources.

## 3 Results

### 3.1 Model Performance and Evaluation

We performed five-fold cross-validation on the training dataset, utilizing 80% of the images for training and 20% for validating the segmentation models. As shown in Fig. S1 in the Supplementary Material, both training and validation curves exhibit stable convergence, with minimal gap between them, suggesting that the model generalized well to unseen data and avoided overfitting. Table 2 presents the mean and standard deviation of the IoU, Dice coefficient, and accuracy for the evaluated models. The results demonstrate that UNet++ with EfficientNet-b7 outperformed the other models, achieving the highest mean IoU of 86.48% (±0.62) and Dice coefficient of 89.87% (±0.37), with an accuracy of 92.75% (±0.35). UNet with EfficientNet-b7 also showed strong performance, whereas TransUNet and Segformer with MiT-b5 displayed slightly lower segmentation performance. Despite these differences, all models demonstrated effective segmentation of CFZs in OCTA images, with clear delineation of arteries, veins, and their corresponding CFZ regions.

**Table 2:** Performance of the deep learning models.

Model	Mean (±std dev)		
	IoU (%)	Dice (%)	Accuracy (%)
UNet (EfficientNet-b7)	85.62 (±0.71)	89.32 (±0.42)	92.25 (±0.39)
UNet++ (EfficientNet-b7)	86.48 (±0.62)	89.87 (±0.37)	92.75 (±0.35)
TransUNet	84.17 (±0.58)	88.70 (±0.33)	91.49 (±0.31)
Segformer (MiT-B5)	82.83 (±0.65)	87.32 (±0.38)	90.68 (±0.34)

**Fig. 2:**
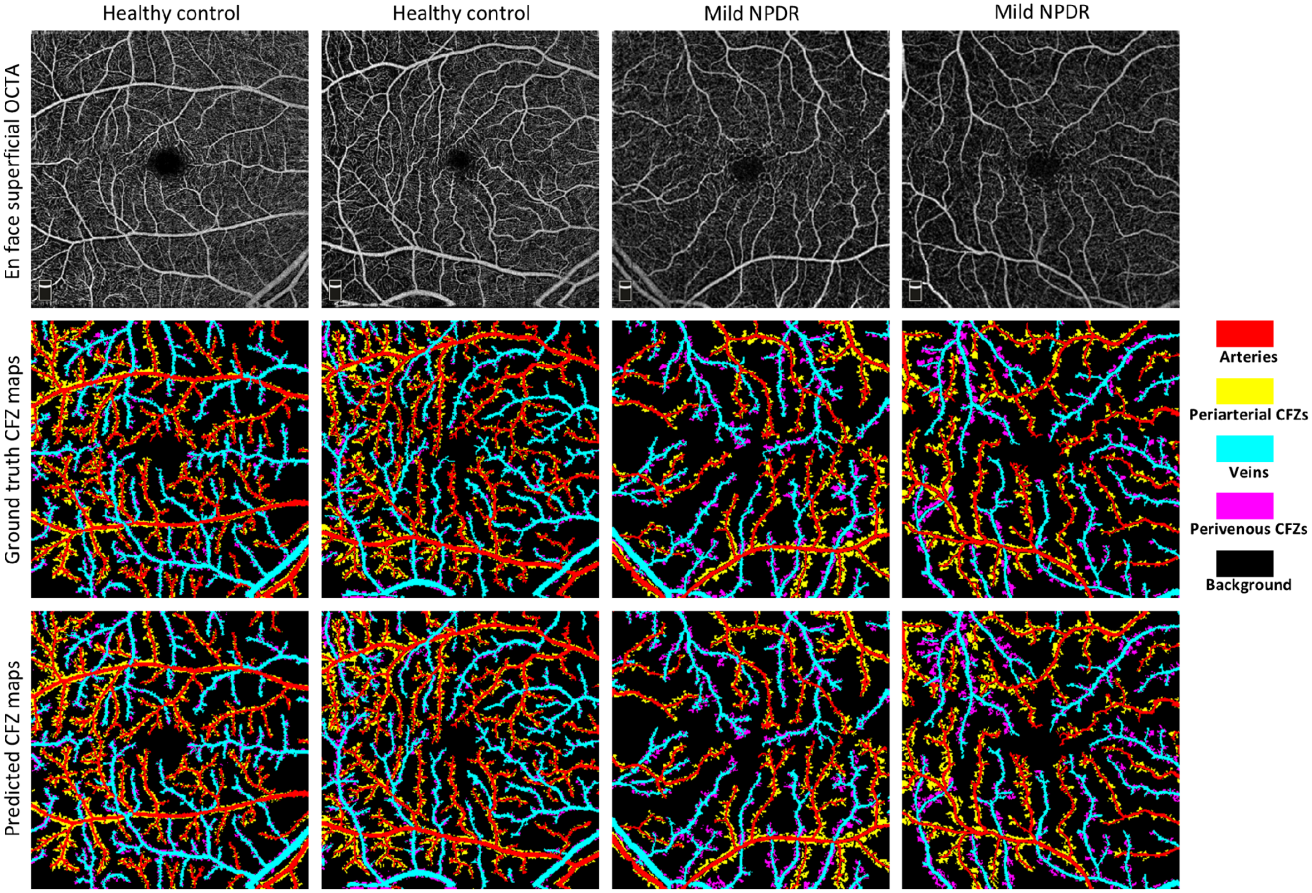
CFZ segmentation of representative OCTA images in the validation dataset. Each column represents a different sample from the validation dataset (two control and two mild NPDR cases). Row 1 shows the original grayscale OCTA images, row 2 shows the ground truth CFZ maps, and row 3 shows the CFZ maps predicted by the best-performing model.

**Fig. 3:**
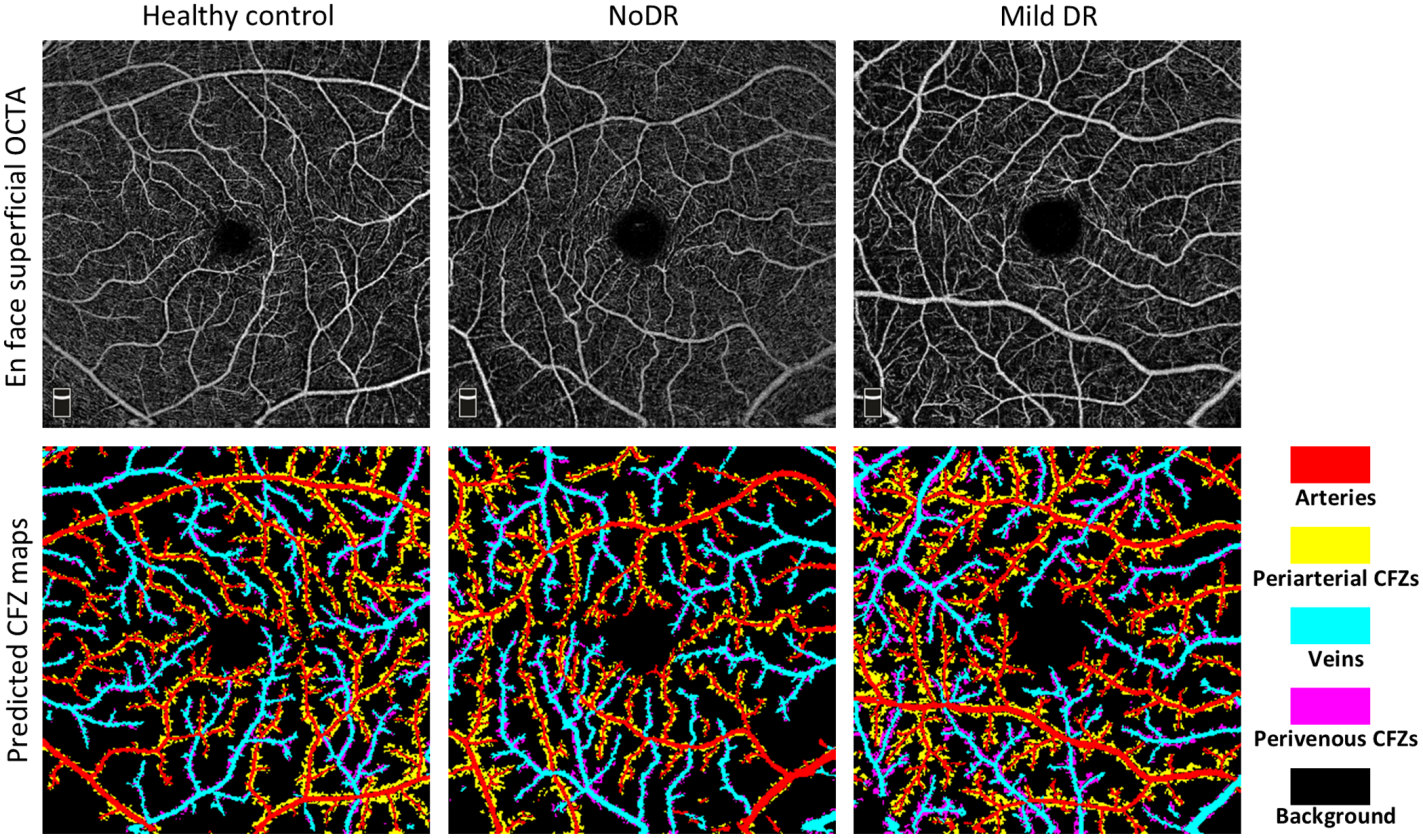
CFZ segmentation of representative OCTA images in the test dataset.

In Fig. 2, we illustrate the visual results of the CFZ segmentation achieved by the best model, UNet++ with EfficientNet-b7, for four validation samples (two control and two mild cases) from the training dataset. The figure displays the grayscale OCTA images, the ground truth CFZ maps, and predicted CFZ maps in the rows from top to bottom, respectively. The results suggest that the predicted CFZ maps for healthy and diseased subjects are highly consistent with the ground truth, demonstrating the models’ ability to accurately detect and classify arteries, veins, and their corresponding periarterial and perivenous CFZs. However, some incorrect segmentations indicate areas for improvement. In addition, Fig. 3 presents the visual performance of the best-performing model for segmenting representative OCTA images in the test dataset. The figure highlights the segmentation of key areas such as arteries, veins, and their corresponding CFZ regions across control, NoDR, and mild DR samples. The model performed well qualitatively in identifying retinal vessels and CFZ areas.

### 3.2 Quantitative Analysis

Studying the test dataset with demographic details presented in Table 1, no statistically significant differences were found between the different groups regarding age and sex (ANOVA, p=0.118; and $\chi^{2}$ test, p=0.509, respectively). The CFZ-based features defined in previous section, including the periarterial CFZ ratio ($R_{a}$), perivenous CFZ ratio ($R_{v}$), total CFZ ratio ($R_{t}$), number of periarterial CFZs ($N_{a}$), number of perivenous CFZs ($N_{v}$), number of total CFZs ($N_{t}$), and their corresponding mean sizes ($M_{a}$, $M_{v}$, $M_{t}$), were calculated for the entire test dataset. These quantitative metrics provide insights into the spatial and structural changes in the CFZ across different stages of DR.

We performed the Mann-Whitney U test, a non-parametric method, to conduct pairwise comparisons between the control, NoDR, and mild DR groups. Significant differences between groups were denoted by p values of <0.05, <0.017, and <0.001, marked by *, **, and ***, respectively. To adjust for multiple comparisons and reduce the risk of false positives, we applied a Bonferroni correction, resulting in an adjusted significance level of p<0.017 for the comparisons among the three groups.

**Fig. 4:**
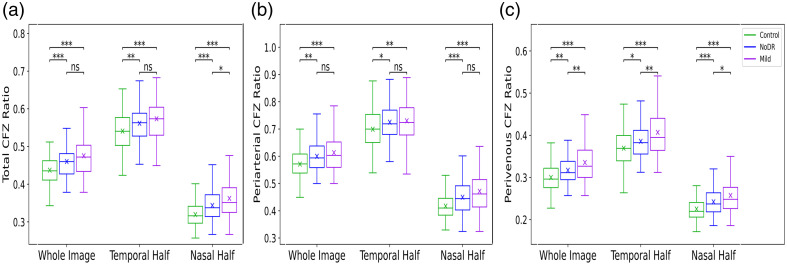
Boxplots of CFZ ratios for control, NoDR, and mild DR subjects. (a) Total CFZ ratio, (b) periarterial CFZ ratio, and (c) perivenous CFZ ratio are shown for the whole image, nasal half, and temporal half.

Figure 4 presents the boxplots of the total CFZ ratio ($R_{t}$), periarterial CFZ ratio ($R_{a}$), and perivenous CFZ ratio ($R_{v}$) for control, NoDR, and mild DR subjects in the whole image, nasal half, or temporal half. The results reveal a general increasing trend in all three CFZ ratios as we move from control to NoDR and then to mild DR groups, indicating progressive microvascular changes as DR advances. Notably, the perivenous CFZ ratio exhibits a more significant change across all three groups compared with the periarterial CFZ ratio and total CFZ ratio.

**Fig. 5:**
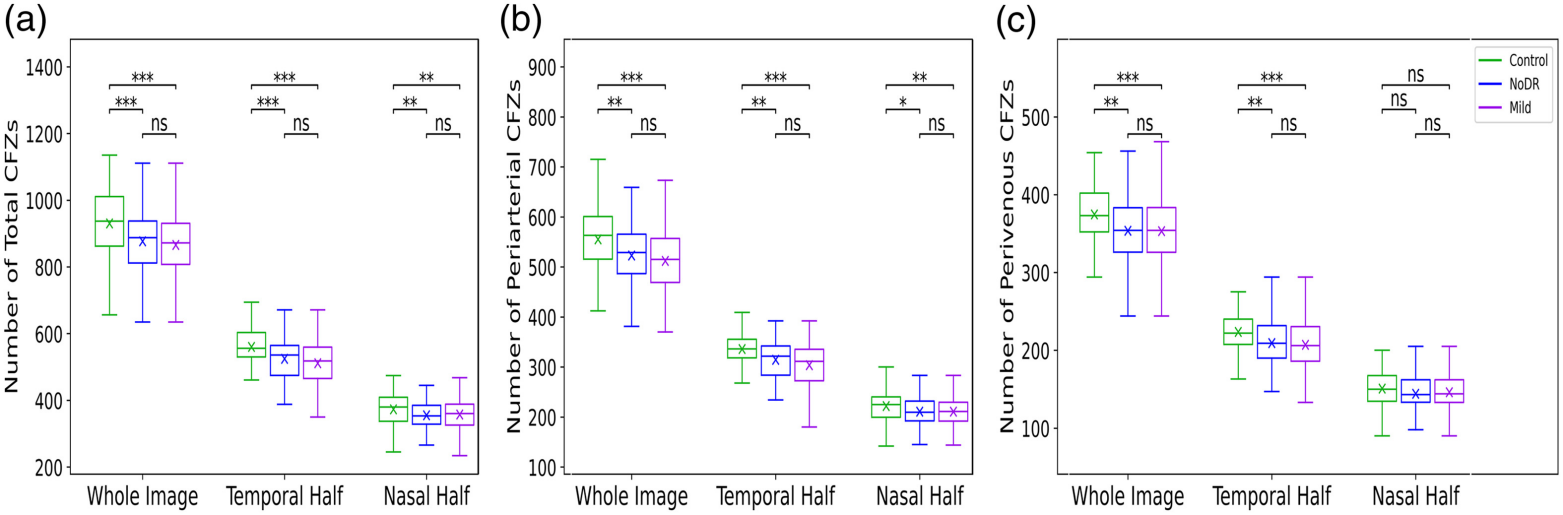
Boxplots of CFZ numbers for control, NoDR, and mild DR subjects. (a) Total CFZ numbers, (b) periarterial CFZ numbers, and (c) perivenous CFZ numbers are shown for the whole image, temporal half, and nasal half.

**Fig. 6:**
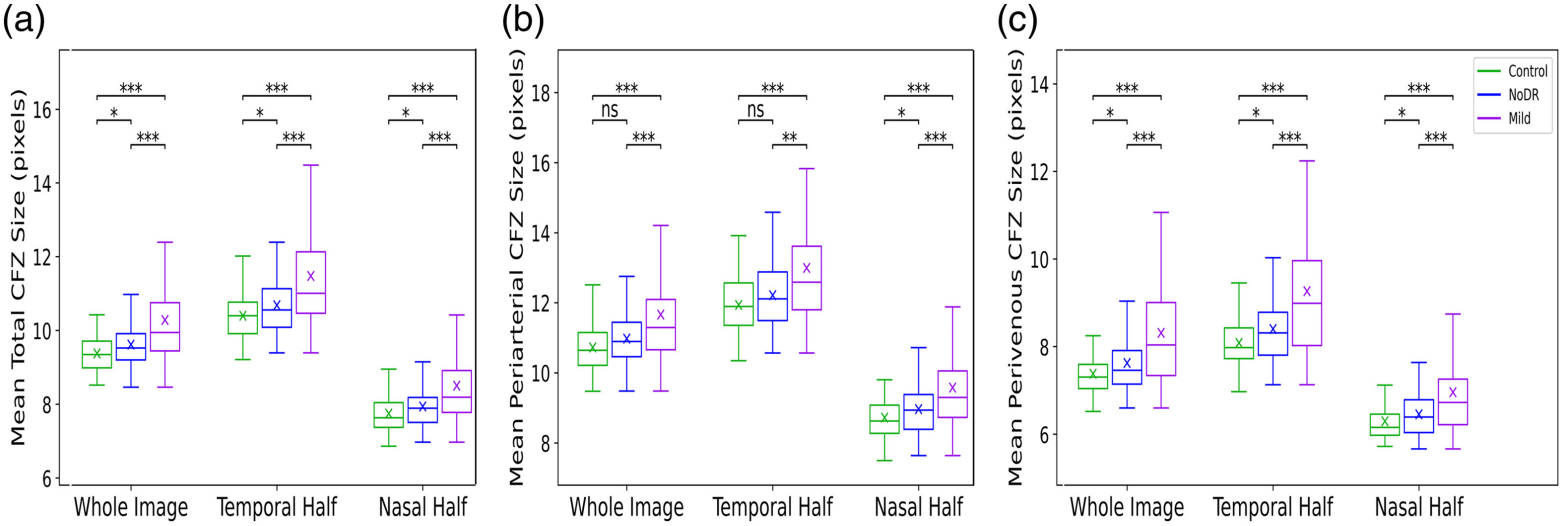
Boxplots of mean CFZ sizes for control, NoDR, and mild DR subjects. (a) Mean total CFZ size, (b) mean periarterial CFZ size, and (c) mean perivenous CFZ size are presented for the whole image, temporal half, and nasal half.

Figure 5 demonstrates the boxplots of the number of CFZs for control, NoDR, and mild DR subjects, showing the total number of CFZs ($N_{t}$), periarterial CFZs ($N_{a}$), and perivenous CFZs ($N_{v}$) in the whole image, nasal half, and temporal half. The results indicate a significant decrease in the number of CFZs across all categories as DR progresses. Specifically, the NoDR and mild DR groups exhibit notable reductions in the total number of CFZs, periarterial CFZs, and perivenous CFZs compared with the control group. This trend is more pronounced in the temporal half of the retina. Figure 6 displays the boxplots of the mean CFZ sizes, including the mean total CFZ size ($M_{t}$), mean periarterial CFZ size ($M_{a}$), and mean perivenous CFZ size ($M_{v}$) for control, NoDR, and mild DR subjects across the whole image, nasal half, and temporal half. The results indicate a clear trend of increasing mean CFZ sizes as DR progresses from control to mild DR stages. This enlargement is particularly noticeable in the mild DR group, where the mean total CFZ size, mean periarterial CFZ size, and mean perivenous CFZ size show a substantial increase compared with the control and NoDR groups. These findings highlight the potential of mean CFZ size as a sensitive biomarker for detecting early microvascular changes in DR.

## 4 Discussion and Conclusion

Early detection of DR relies heavily on the analysis of capillaries because diabetes primarily affects the microvasculature in its initial stages.^35^ One of the earliest cellular changes in diabetic capillaries is the loss of pericytes, which are vital cells that help regulate blood flow, maintain capillary stability, and prevent microaneurysm formation.^36^ This pericyte loss disrupts the blood-retinal barrier, leading to increased susceptibility to vascular damage.^37^ Alongside these changes, endothelial cell dysfunction occurs, contributing to the breakdown of the blood-retinal barrier, increased vascular permeability, and initiating inflammation that exacerbates vascular instability.^38^ As these processes progress, the capillaries weaken, becoming more susceptible to occlusion and leakage. Together, these changes lead to early capillary nonperfusion, where some capillaries shut down, resulting in localized ischemia, hypoxia, and reduced oxygen supply in the retinal tissue.^39^ These changes can contribute to the enlargement or alteration of periarterial and perivenous CFZs.

This underscores the importance of using quantitative analysis of periarterial and perivenous CFZs for evaluating DR, especially in its early stages, as well as other retinal conditions that impact the microvasculature. In DR, the enlargement or irregularity of these CFZs serves as an early indicator of microvascular dysfunction. By accurately measuring these regions, clinicians can detect subtle capillary dropout and nonperfusion, which are critical markers of disease progression. Such precise analysis allows for earlier diagnosis and intervention. Moreover, this approach could be valuable in assessing other retinal diseases, such as hypertensive retinopathy or retinal vein occlusions. The advent of OCTA offers a noninvasive imaging modality that provides detailed visualization of retinal capillary networks and blood flow, enabling clinicians to detect subtle changes in the microvasculature with high precision. OCTA offers a noninvasive method to quantify periarterial and perivenous CFZs, providing significant advantages in retinal vascular imaging.^40^ According to recent findings comparing OCTA to histology, OCTA’s capability to measure periarterial and perivenous CFZs has proven invaluable in identifying early microvascular alterations that precede the clinical onset of DR.^40^ Segmenting and distinguishing between periarterial and perivenous CFZs automatically allows for a more targeted assessment of capillary dropout and ischemia, which are early indicators of retinal damage in diabetic patients.

In this study, we employed deep learning models to segment periarterial and perivenous CFZs from OCTA images, creating detailed CFZ maps that offered critical information about the retinal vasculature. We utilized state-of-the-art architectures, including UNet, UNet++, TransUNet, and Segformer, to achieve high-precision segmentation. Among these, UNet++ with EfficientNet-b7 demonstrated the highest performance, achieving a mean IoU of 86.48%, Dice coefficient of 89.87%, and accuracy of 92.75%, indicating its superior ability to preserve spatial details and deliver precise segmentation outcomes. The CFZ maps generated by the best-performing model enabled us to quantitatively assess changes in the CFZs by calculating metrics such as CFZ ratios, CFZ counts, and mean CFZ sizes for total, periarterial, and perivenous regions. These quantitative features allowed us to quantify and analyze the relative changes in vascular characteristics between control, NoDR, and mild NPDR groups, facilitating the early detection of DR.

The results illustrated in Fig. 4 demonstrate a general increasing trend in all three CFZ ratios as we move from the control to NoDR and then to mild groups. Significant increases are observed in the total, periarterial, and perivenous CFZ ratios in the NoDR or mild groups compared with the control group. However, the change in the total CFZ ratio between NoDR and mild is not significant, particularly for the whole image and the temporal half. Notably, when analyzing the periarterial and perivenous CFZs separately, the perivenous CFZ ratio shows significant increases from NoDR to mild in the whole image, temporal half, and nasal half, highlighting its potential as a quantitative feature that can differentiate among all three groups. The results shown in Fig. 5 reveal a general decreasing trend in the number of total, periarterial, and perivenous CFZs as we progress from the control group to NoDR and then to the mild group. Significant reductions are observed in the number of these CFZs in both the NoDR and mild groups compared with the control group. However, the decreases in the total CFZ count between NoDR and mild are not significant. In addition, the perivenous CFZ count in the nasal half shows no significant differences between the three groups.

In Fig. 6, we observe a general increasing trend in the mean CFZ sizes across all three categories—total, periarterial, and perivenous CFZ sizes—as we move from the control group to NoDR and then to the mild group. Highly significant increases in mean total CFZ size, periarterial CFZ size, and perivenous CFZ size are observed in the mild group compared with both the control and NoDR groups. Notably, changes in mean total CFZ size between the control and NoDR groups are significant for the total and perivenous CFZs but not for the periarterial CFZs, particularly in the whole image and the temporal half. This suggests that mean CFZ size may serve as a highly effective quantitative feature or biomarker, showing significant changes across all three groups and playing a crucial role in the early detection of DR.

The quantitative features introduced in this study underscore their potential for early detection of DR by capturing CFZ changes across various stages. Even with Bonferroni correction applied, the significant alterations in CFZ ratios and CFZ counts across all categories (total, periarterial, and perivenous) from control to NoDR groups, as well as from control to mild groups, highlight their effectiveness as biomarkers in distinguishing these stages. In addition, the mean CFZ size consistently shows significant increases across all categories as DR progresses from control to NoDR and from NoDR to mild stages, emphasizing its value as a sensitive quantitative feature for early DR detection. The combined use of CFZ ratios, CFZ counts, and mean CFZ size provides a comprehensive approach that leverages these CFZ-based quantitative features to enhance the accuracy and robustness of DR detection and classification, as well as the assessment of other retinal pathologies.

Although our multicenter study enhances the validity of our findings across diverse populations and clinical settings, the limited dataset size is a limitation of this study; therefore, for some subjects, both eyes were included in the statistical analysis of quantitative features. There may be some correlation between right and left eyes due to genetics and environmental factors. Our findings lay the groundwork for future research, including the application of CFZ-based quantitative analysis to other retinal diseases, as well as systematic investigations into the effects of OCTA image quality on segmentation performance. In addition, future studies can aim to validate and adapt the proposed models using scans acquired from different OCTA devices, to assess and enhance cross-platform generalizability. Although our U-Net++–based model performs well, further improvements are possible; for example, incorporating attention mechanisms or transformer-based modules could help the network capture long-range context, potentially refining CFZ edge detection.

In conclusion, this study introduced deep learning models for the segmentation of periarterial and perivenous CFZs in OCTA images. The deep learning models, particularly the UNet++ with EfficientNet-b7 backbone, demonstrated robust performance, highlighting the efficacy of using advanced architectures for precise periarterial and perivenous CFZ segmentation. The quantitative features derived from CFZ analysis, including CFZ ratios, counts, and mean sizes, successfully distinguished control, NoDR, and mild DR stages. The significant variances in these features across groups underscore their potential as biomarkers for early detection of DR. This work establishes a foundation for automated CFZ analysis, with promising applications beyond DR to other retinal diseases, potentially enhancing early diagnosis and disease monitoring.

## Supplementary Material

10.1117/1.JBO.30.5.056005.s01

## Data Availability

The code used to generate the results and figures is available in two GitHub repositories [https://github.com/mansour2002/CFZ-Net-training] and [https://github.com/mansour2002/TransUnet-for-CFZ-Segmentation]. Data are not publicly available. They may be obtained from the authors upon reasonable request.
