# Supplementary material for: Deep learning segmentation of periarterial and perivenous capillary-free zones in optical coherence tomography angiography
Source: J Biomed Opt. 2025 May 8;30(5):056005. doi: 10.1117/1.JBO.30.5.056005 (PMC12061543; doi:10.1117/1.JBO.30.5.056005)
Supplement: Supplementary file 1 [file JBO_030_056005_SD001.pdf]

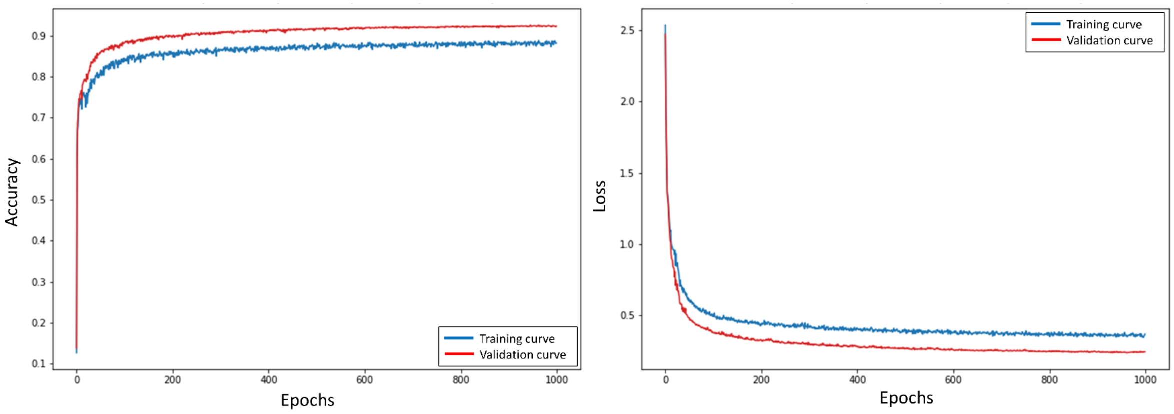

Figure S1. Learning curves of training and validation accuracy and loss over 1000 epochs for the best-performing model (UNet++ with EfficientNet-b7).
